# A Wild Bootstrap approach for the selection of biomarkers in early diagnostic trials

**DOI:** 10.1186/s12874-015-0025-y

**Published:** 2015-05-01

**Authors:** Antonia Zapf, Edgar Brunner, Frank Konietschke

**Affiliations:** Department of Medical Statistics, University Medical Center Göttingen, Humboldtallee 32, Göttingen, 37073 Germany; Department of Mathematical Sciences, The University of Texas at Dallas, 800 W Campbell Road, Richardson, 75080 TX USA

**Keywords:** AUC, Diagnostic study, Resampling, Simultaneous intervals, Wild bootstrap

## Abstract

**Background:**

In early diagnostic trials, particularly in biomarker studies, the aim is often to select diagnostic tests among several methods. In case of metric, discrete, or even ordered categorical data, the area under the receiver operating characteristic (ROC) curve (denoted by AUC) is an appropriate overall accuracy measure for the selection, because the AUC is independent of cut-off points.

**Methods:**

For selection of biomarkers the individual AUC’s are compared with a pre-defined threshold. To keep the overall coverage probability or the multiple type-I error rate, simultaneous confidence intervals and multiple contrast tests are considered. We propose a purely nonparametric approach for the estimation of the AUC’s with the corresponding confidence intervals and statistical tests. This approach uses the correlation among the statistics to account for multiplicity. For small sample sizes, a Wild-Bootstrap approach is presented. It is shown that the corresponding intervals and tests are asymptotically exact.

**Results:**

Extensive simulation studies indicate that the derived Wild-Bootstrap approach keeps and exploits the nominal type-I error at best, even for high accuracies and in case of small samples sizes. The strength of the correlation, the type of covariance structure, a skewed distribution, and also a moderate imbalanced case-control ratio do not have any impact on the behavior of the approach. A real data set illustrates the application of the proposed methods.

**Conclusion:**

We recommend the new Wild Bootstrap approach for the selection of biomarkers in early diagnostic trials, especially for high accuracies and small samples sizes.

**Electronic supplementary material:**

The online version of this article (doi:10.1186/s12874-015-0025-y) contains supplementary material, which is available to authorized users.

## Background

The aim of early diagnostic trials, particularly of biomarker studies, is often to select the most promising markers from a candidate set. For convenience, all different kinds of diagnostic tests, e.g., imaging techniques or biomarkers, will be denoted by *diagnostic tests* throughout the paper. In these studies, response variables are often not binary, but measured on a continuous, discrete or even ordinal scale and a cut-off value *c* has not yet been chosen. Therefore, the sensitivity (i.e. true positive proportion) and the specificity (true negative proportion) both being computed based on *c* cannot be used as selection criteria. In contrast, the Receiver Operating Characteristic (ROC) curve illustrates the overall diagnostic performance because it is independent of the chosen cut-off values (see, e.g., DeLong, DeLong and Clark-Pearson [1]). Because the ROC curve of a diagnostic test is invariant with respect to any monotone transformation of the test measurement scale, it is an adequate measure for comparing diagnostic tests being measured even on different scales. The Area Under the ROC-curve (AUC) represents an accuracy measure which is independent from the selected cut-off value *c* and which is invariant under any monotone transformation of the data. Therefore, it is an appropriate selection criterion for promising diagnostic tests, and in particular Xia et al. [2] (p. 286) state in their tutorial about translational biomarker discovery in clinical metabolomics that the *“AUC is widely used for performance comparison across different biomarker models”*.

**Figure 1:**
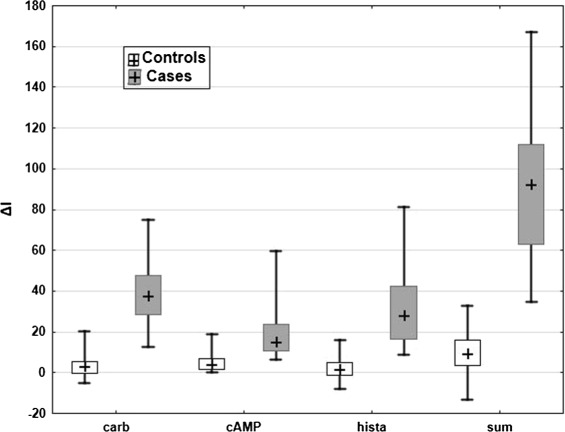
Boxplots of the biomarkers. Boxplot of the four biomarkers in the example, separately for cases and controls.

As an example for the evaluation of different biomarkers we consider the ICM trial by Derichs et al. [3], which aims to evaluate the diagnostic accuracy of intestinal current measurement (ICM) with regard to questionable cystic fibrosis (CF). This study was conducted with the approval of the local ethics committee, MH Hannover, Germany and all patients and/or parents and healthy controls gave their written informed consent. In this trial, a total of *N*=67 children and adults were enrolled. The true disease state of the patients was defined by a composite gold standard, which consists of typical CF symptoms plus either a positive sweat test and/or gene mutations. By this definition 26 patients were classified into CF (referred to as cases) and 41 into ‘CF unlikely’ (referred to as controls). Furthermore, four biomarkers were considered: *ΔI*_*sc*,*carbachol*_, *ΔI*_*sc*,*cAMP*/*forskolin*_, and *ΔI*_*sc*,*histamine*_ (abbreviated by *ΔI*_*carb*_, *ΔI*_*cAMP*_, and *ΔI*_*hista*_) as well as the sum of the three measured values, *ΔI*_*sum*_. Boxplots of the data are displayed in Figure 1.

**Figure 2:**
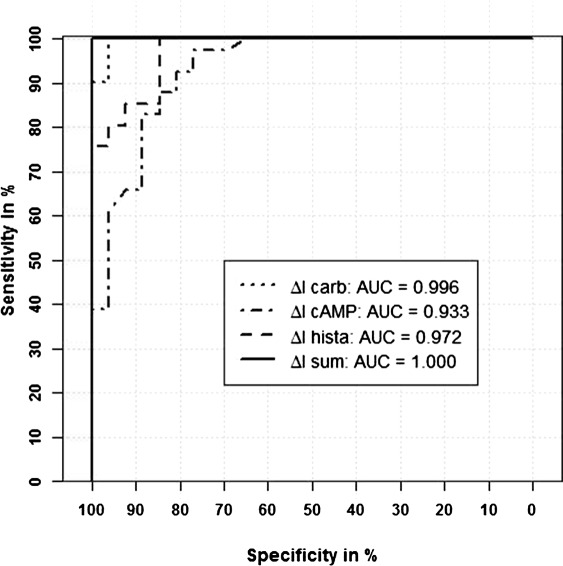
ROC-curves of the biomarkers. ROC-curves of the four biomarkers in the example and corresponding AUC’s in the legend.

In the ROC-curves in Figure 2 the corresponding estimated AUC’s are added. It can be readily seen that the diagnostic accuracy of *ΔI*_*carb*_, *ΔI*_*cAMP*_, and *ΔI*_*hista*_ is quite good, and that *ΔI*_*sum*_ perfectly differentiates the cases and the controls.

Thus, the remaining question is which biomarkers have sufficient diagnostic accuracy. There is no consensus about the threshold for sufficent diagnostic accuracy. Xia et al. [2] characterize a biomarker with an *AUC*<0.7 as a quite “weak” biomarker. In their study about a blood-based biomarker panel for stratifying current risk for colorectal cancer Marshall et al. [4] accept a candidate model with an *AUC*>0.75 as a predictive model. In contrast, Broadhurst and Kell [5] refer to an *AUC*>0.9 as excellent and to an *AUC*>0.8 as good. Depending on previous knowledge or expectations a threshold for the AUC as indicator for sufficient diagnostic accuracy should be chosen during the planning of the trial.

Note that the aim of such trials is not to test multiple hypotheses formulated in terms of AUC differences across the biomarkers, but to verify sufficient diagnostic accuracy for all biomarkers individually. Then comparing the lower limit of the confidence interval for the estimated AUC with this threshold indicates whether or not the diagnostic test has sufficient diagnostic accuracy. The “Guideline on the choice of the non-inferiority margin” of the European Medicines Agency [6] recommends to demonstrate non-inferiority by use of two-sided 95*%* or one-sided 97.5*%* confidence intervals.

If several diagnostic tests are evaluated in the same trial, it is important to adjust the confidence intervals for multiplicity. Otherwise there is a high risk that the accuracy of some diagnostic tests is overestimated. Xia et al. [2] (p.288) point out that *“The probability of finding a random association between a given metabolite and the outcome increases with the total number of comparisons”*. Furthermore they note that the Bonferroni correction is a simple but very conservative method. If the diagnostic tests are repeatedly measured on the same subjects, hence, these measurements are correlated in general. Therefore it is of highly practical importance to take into account these correlations in the estimation of the diagnostic accuracy.

The multiplicity expert group of the ‘Statisticians in the Pharmaceutical Industry’ [7] (p.258) states that *“The participants did, however, agree that for non-inferiority and equivalence trials, compatible simultaneous CIs for the primary endpoint(s) should be presented in all cases”*. Furthermore Strassburger and Bretz [8] recommend the use of single-step procedures if the aim is not to reject as many hypotheses as possible. Therefore we will confine ourselves to simultaneous confidence intervals from single-step procedures which are compatible with the results obtained by hypotheses tests. Among others, Hothorn et al. [9] proposed parametric simultaneous confidence intervals, which correspond to multiple contrast tests. However, since these parametric approaches are limited to normally distributed data, Konietschke et al. [10] proposed nonparametric multiple contrast tests and compatible asymptotic simultaneous confidence intervals for relative treatment effects for independent samples (based on some theoretical results developed by Brunner et al. [11]). In the particular case of two samples (cases and controls) the relative treatment effect is equivalent to the AUC (see Bamber [12]). In this article we will use this approach in the framework of diagnostic studies, but for paired samples in a multivariate layout.

The challenge in early diagnostic trials is often that smaller sample sizes and higher AUC’s occur. For example in the systematic review of 10 studies about the diagnostic accuracy of pleural fluid NT-pro-BNP for pleural effusions of cardiac origin, performed by Janda and Swiston [13], the median total sample size was 104 (mean 112), and the pooled AUC was 98*%*. Wang et al. [14] reported in another systematic review about cardiac testing for coronary artery disease in potential kidney transplant recipients AUC’s between 0.78 and 0.92. Kottas et al. [15] found that the Logit tranformation based confidence interval for a single AUC leads to slightly conservative results for small sample sizes. Here we suggest Wild Bootstrap based simultaneous confidence intervals to obtain robust methods for small sample sizes and potentially quite large AUC’s. Hereby, we generalize the method proposed by Arlot et al. [16] for multivarite high-dimensional normal data.

In this article nonparametric simultaneous confidence intervals for multiple AUC’s in diagnostic studies are presented. Asymptotic intervals will be derived as well as intervals using the Wild Bootstrap approach. The properties of these simultaneous intervals are investigated in a simulation study regarding the type-I error rate and the statistical power. Furthermore, the results of all intervals are given for the example data set presented before in this section. In the next section we present the methods, including the statistical model with the corresponding hypotheses, and the point estimators with their asymptotic distribution. Furthermore multiple contrast tests and corresponding simultaneous confidence intervals (with or without Logit transformation) are derived, and the Wild Bootstrap approach is presented (in particular for small sample sizes). The results of a simulation study including robustness evaluations, and the application of the methods to the example presented above are given in the Results section. Finally, all results are summarized and discussed, and a recommendation is given.

## Methods

### Statistical model and hypotheses

We consider a within-subject multi-modality diagnostic trial given by independent and identically distributed random vectors

(1) $$ {} \begin{aligned} \mathbf{X}_{is}=\left(X_{is}^{(1)},\ldots,X_{is}^{(d)}\right)' \sim \mathbf{F}_{i}, \;& i=0,1 \text{(control, case)}; \,\\ &{\kern-2.7pc} \text{subject }s=1,\ldots,n_{i}, \end{aligned}  $$

with marginal distributions

(2) $$\begin{array}{@{}rcl@{}} X_{is}^{(\ell)}\sim F_{is}^{(\ell)}, \ell=1,\ldots,d, \end{array} $$

where *d* denotes the number of diagnostic tests. The partition of the data in cases (*i*=1) or controls (*i*=0) is based on the gold or reference standard, which is assumed to represent the true disease status of the subjects. In order to allow for continuous, discrete or even ordered categorical data in a unified way, we use the normalized version of the marginal distribution functions, i.e., $F_{i}^{(\ell)}(x) = \tfrac 12\left (F_{i}^{(+,\ell)}(x) + F_{i}^{(-,\ell)}(x)\right)$, where $F_{i}^{(+,\ell)}(x) = P\left (X_{i1}^{(\ell)}\leq x \right)$ denotes the right-continuous and $F_{i}^{(-,\ell)}(x) = P\left (X_{i1}^{(\ell)}< x\right)$ denotes the left-continuous version of the distribution function respectively. In the context of nonparametric models, the normalized version of the distribution function was first mentioned by Kruskal [17] and generally dates back to Lévy [18]. Later on, it was used by Ruymgaart [19], Munzel [20], Brunner and Puri [21], Kaufmann et al. [22], among others, to derive asymptotic results for rank statistics including the case of ties. We note that $F_{i}^{(\ell)}(x)$ may be arbitrary distribution functions, with the exception of the trivial case that both distributions are one-point distributions (see Lange and Brunner [23]).

The within-subject design given in (1), which means that all diagnostic tests are performed in each individual, is recommended in the EMA guideline about diagnostic agents [24] and refers to Design 1 in Brunner and Zapf [25].

For each of the *d* diagnostic tests the true AUC is given by

(3) $$ \begin{aligned} AUC^{(\ell)}&=P(X_{01}^{(\ell)}<X_{11}^{(\ell)})+0.5\cdot P(X_{01}^{(\ell)}=X_{11}^{(\ell)})\\&=\int{ F_{0}^{(\ell)}{dF}_{1}^{(\ell)}}\;, \ell = 1,\ldots,d. \end{aligned}  $$

For a convenient derivation of asymptotic results, the *AUC*’s are collected in the vector **AUC** = (*AUC*^(1)^, …,*AUC*^(*d*)^)^′^.

In order to select the most promising diagnostic tests from the candidate set of the *d* different methods, it is our aim to test the non-inferiority null hypotheses

(4) $$  \begin{aligned} &H_{0}: \bigcap_{\ell=1}^{d} \left\{H_{0}^{(\ell)}:AUC^{(\ell)} \leq {AUC}_{0}\right\} \;\; \; \text{versus}\; \; \; \\&H_{1}: \bigcup_{\ell=1}^{d} \left\{H_{1}^{(\ell)}:AUC^{(\ell)} > {AUC}_{0}\right\} \end{aligned}  $$

with strong control of the familywise error rate (FWER) *α* simultaneously. The non-inferiority margin *AUC*_0_ is assumed to have been fixed during the planning phase of the trial. Thus, the set of promising diagnostic tests consists of all markers, whose corresponding *AUC*^(*ℓ*)^ have been declared to be larger than *AUC*_0_ by an adequate multiple testing procedure.

### Point estimators and asymptotic distribution

Unbiased and *L*_2_-consistent point estimators for the AUC’s defined in (3) are derived by replacing the unknown distribution functions $F_{0}^{(\ell)}$ and $F_{1}^{(\ell)}$ by their empirical counterparts

$$\begin{array}{@{}rcl@{}} \widehat{F}_{i}^{(\ell)}(x) = \frac{1}{n_{i}} \sum\limits_{s=1}^{n_{i}} c(x-X_{is}^{(\ell)}), \; i=0,1; \; \ell = 1,\ldots,d, \end{array} $$

where *c*(*x*) denotes the normalized version of the count function, i.e. $c(x) \in \{0,\tfrac 12,1\}$ corresponding to {*x*<0,*x*=0,*x*>0}, respectively. The point estimator

(5) $$ \widehat{AUC}^{(\ell)}=\int{\widehat{F}_{0}^{(\ell)}d\widehat{F}_{1}^{(\ell)}}=\frac{1}{N}\left(\overline{R}_{1.}^{(\ell)}-\overline{R}_{0.}^{(\ell)}\right)+\frac{1}{2}  $$

can easily be computed using the means $\overline {R}_{i.}^{(\ell)} = n_{i}^{-1} \sum _{s=1}^{n_{i}} R_{\textit {is}}^{(\ell)}$ of the (mid-) ranks $R_{\textit {is}}^{(\ell)}$, *i*=0,1. Here, $R_{\textit {is}}^{(\ell)}$ denotes the rank of $X_{\textit {is}}^{(\ell)}$ among all *N*=*n*_0_+*n*_1_ observations $X_{01}^{(\ell)}, \ldots, X_{0n_{0}}^{(\ell)}$, $ X_{11}^{(\ell)}, \ldots, X_{1n_{1}}^{(\ell)}$ per marker *ℓ*=1,…,*d*. Further let $\mathbf {R}_{\textit {is}}=\left (R_{\textit {is}}^{(1)},\ldots,R_{\textit {is}}^{(d)}\right)'$ denote the vectors of the midranks and let $\widehat {\mathbf {AUC}}=\left (\widehat {AUC}^{(1)},\ldots,\widehat {AUC}^{(d)}\right)'$ denote the vector of the point estimators.

Brunner et al. [11] have shown that the vector $\sqrt {N}(\widehat {\mathbf {AUC}} - \mathbf {AUC}) $ follows, asymptotically, as *N*→*∞*, a multivariate normal distribution with expectation **0** and covariance matrix

(6) $$ \mathbf{V}_{N} = Cov(\sqrt{N}\mathbf{B}),  $$

where **B**=(*B*^(1)^,…,*B*^(*d*)^)^′^ denotes a random vector the components of which are sums of independent random variables

(7) $${} {\small{\begin{aligned} B^{(\ell)}=\frac{1}{n_{1}}\sum\limits_{s=1}^{n_{1}}F_{0}^{(\ell)}(X_{1s}^{(\ell)})-\frac{1}{n_{0}}\sum\limits_{s=1}^{n_{0}}F_{1}^{(\ell)}(X_{0s}^{(\ell)})+1-2\cdot AUC^{(\ell)}. \end{aligned}}}  $$

The covariance matrix **V**_*N*_ with elements *v*^(*ℓ*,*m*)^, however, is unknown and has to be estimated. Let $R_{\textit {is}}^{(i|\ell)}$ denote the so-called internal rank of $X_{\textit {is}}^{(\ell)}$ among all *n*_*i*_ observations $X_{i1}^{(\ell)},\ldots, X_{{in}_{i}}^{(\ell)}$ for the diagnostic test *ℓ* in disease status group *i*, and let $\mathbf {R}_{\textit {is}}^{(i)}=\left (R_{\textit {is}}^{(i|1)},\ldots,R_{\textit {is}}^{(i|d)}\right)'$ denote the vectors of these internal ranks. Furthermore, let

(8) $$\begin{array}{@{}rcl@{}}  \mathbf{Z}_{is} = \frac{1}{N-n_{i}} \left(\mathbf{R}_{is} - \mathbf{R}_{is}^{(i)}\right) \end{array} $$

denote the vectors of the normed placements

$$\begin{aligned} &\widehat{F}_{0}^{(\ell)}(X_{1s}^{(\ell)}) = \frac{1}{n_{0}} \left(R_{1s}^{(\ell)} - R_{1s}^{(1|\ell)} \right) \; \text{and} \; \\&\widehat{F}_{1}^{(\ell)}(X_{0s}^{(\ell)}) = \frac{1}{n_{1}} \left(R_{0s}^{(\ell)} - R_{0s}^{(0|\ell)} \right), \end{aligned} $$

respectively. Then a consistent estimator of the covariance matrix is given by $\widehat {\mathbf {V}}_{N}=N \left (\widehat {\mathbf {V}}_{N,0}/n_{0}+\widehat {\mathbf {V}}_{N,1}/n_{1} \right)$, where

(9) $${} \widehat{\mathbf{V}}_{N,i}=\frac{1}{n_{i}-1}\sum\limits_{s=1}^{n_{i}}\left(\mathbf{Z}_{is}-\overline{\mathbf{Z}}_{i.}\right)\left(\mathbf{Z}_{is}-\overline{\mathbf{Z}}_{i.}\right)'\,,\,i=0,1.  $$

Here, $ \overline {\mathbf {Z}}_{i\cdot } = \frac {1}{n_{i}} \sum _{s=1}^{n_{i}}\mathbf {Z}_{is}$ denotes the vector of means of the normed placements. For more details we refer to Brunner et al. [11] and Kaufmann et al. [22].

### Test statistics and confidence intervals

In order to test the null hypotheses formulated in (4), we first need to derive an univariate test statistic for testing the individual null hypothesis $H_{0}^{(\ell)}: AUC^{(\ell)} \leq AUC_{0}$. It follows from the asymptotic multivariate normality of the vector $\sqrt {N}(\widehat {\mathbf {AUC}} - \mathbf {AUC})$ that $\sqrt {N}(\widehat {AUC}^{(\ell)} - AUC^{(\ell)})$ has, asymptotically as *N*→*∞*, a univariate normal distribution with mean 0 and variance *v*^(*ℓ*,*ℓ*)^, i.e. *N*(0,*v*^(*ℓ*,*ℓ*)^). Here, *v*^(*ℓ*,*ℓ*)^ denotes the *ℓ*-th diagonal element of **V**_*N*_ in (6). Hence, by Slutzky’s theorem, it follows that

(10) $${} {\small{\begin{aligned} T^{(\ell)} = \left(\widehat{AUC}^{(\ell)} - AUC^{(\ell)}\right)\sqrt{\frac{N}{\widehat{v}^{(\ell,\ell)}}} \stackrel{\mathcal{D}}{\to} N(0,1), \; \text{as} \; N \to \infty, \end{aligned}}}  $$

where $\widehat {v}^{(\ell,\ell)}$ denotes the diagonal elements of $\widehat {V}_{N}$, defined in (9). In particular, each statistic is studentized with an individual consistent variance estimator and thus, the set of hypotheses and test statistics $\mathbf {\Omega } = \left \{ \left (H_{0}^{(\ell)}, T^{(\ell)}\right), \ell =1,\ldots,d \right \}$ constitutes a joint-testing family in the sense of Gabriel [26]. Attention should be paid to the fact that the estimated variance $\widehat {v}^{(\ell,\ell)}$ is equal to zero if $\widehat {AUC}^{(\ell)}=0$ or 1. Thus, the test statistic *T*^(*ℓ*)^ can not be computed. One possibility to solve this problem is to modify the data slightly (see the analysis of the example in the Results section).

A quite conservative selection approach can be derived by applying the Bonferroni method (denoted as ‘Bonf’), i.e., the individual null hypothesis $H_{0}^{(\ell)}: AUC^{(\ell)} \leq {AUC}_{0}$ will be rejected at multiple level *α*, if *T*^(*ℓ*)^≤*z*_1−*α*/*d*,1_, where *z*_1−*α*/*d*,1_ denotes the one-sided (1−*α*/*d*)-quantile of the standard normal distribution. Asymptotic one-sided simultaneous confidence intervals for the treatment effects *AUC*^(*ℓ*)^ are then given by

(11) $$ {CI}_{Bonf}^{(\ell)}=\left[\widehat{AUC}^{(\ell)}- z_{1-\alpha/d,1}\sqrt{\tfrac{\widehat{v}^{(\ell,\ell)}}{N}} ; 1\right]\,.  $$

The global null hypothesis *H*_0_:**AUC**≤*AUC*_0_·**1** as defined in (4) will be rejected, if max{*T*^(1)^,…,*T*^(*d*)^}>*z*_1−*α*/*d*,1_ or, equivalently, if the maximum of the lower limits of the confidence intervals $\max \{{CI}_{Bonf,l}^{(1)},\ldots, {CI}_{Bonf,l}^{(d)}\} > {AUC}_{0}$. Here ***1***=(1,…,1)^′^ denotes a *d*-dimensional vector of 1s. The Bonferroni method is, however, a quite conservative selection approach (see Results section for more details). The reason for this is that the apparent correlations among the different pivotal quantitites *T*^(1)^,…,*T*^(*d*)^ are not taken into account by this method.

#### Multiple contrast tests and simultaneous confidence intervals

In order to use the correlation in the selection approach, it is our idea to apply the multiple contrast test principle (denoted by MCP), which uses the correlation among different test statistics. The key point of these procedures is to use the joint distribution of a set of statistics to adjust for multiplicity. Thus, the asymptotic multivariate distribution of the vector **T**=(*T*^(1)^,…,*T*^(*d*)^)^′^ is required. The details are stated in the next theorem.

##### **Theorem1**.

Under the assumption that *N*→*∞* such that *N*/*n*_*i*_≤*N*_0_<*∞*, *i*=0,1, the vector **T** follows, asymptotically, a multivariate normal distribution with expectation **0** and correlation matrix **R**, where **R**=[*r*^(*ℓ*,*m*)^]_*ℓ*,*m*=1…,*d*_, and $r^{(\ell,m)} = \tfrac {v^{(\ell,m)}}{\sqrt {v^{(\ell,\ell)} v^{(m,m)}}}$.

The joint distribution of **T** can be used for the derivation of a simultaneous test procedure. Let *z*_1−*α*,1_(**R**) denote the one-sided (1−*α*) equicoordinate quantile of the multivariate normal distribution with expectation ***0*** and correlation matrix **R**, i.e., *N*(**0**,**R**), that is

$$\begin{array}{@{}rcl@{}} P\left(\bigcap\limits_{\ell=1}^{d} \left\{T^{(\ell)}\leq z_{1-\alpha,1}(\mathbf{R})\right\}\right) = 1- \alpha. \end{array} $$

For details see Bretz et al. [27]. Then, the individual null hypothesis $H_{0}^{(\ell)} AUC^{(\ell)} \leq {AUC}_{0}$ will be rejected at multiple level *α*, if

(12) $$\begin{array}{@{}rcl@{}} T^{(\ell)}\geq z_{1-\alpha,1} (\mathbf{R}). \end{array} $$

Asymptotic one-sided simultaneous confidence intervals for *AUC*^(*ℓ*)^ are given by

(13) $$ {CI}_{MCP}^{(\ell)}=\left[\widehat{AUC}^{(\ell)}- z_{1-\alpha,1}(\mathbf{R})\sqrt{\tfrac{\widehat{v}^{(\ell,\ell)}}{N}} ; 1\right]\;.  $$

The global null hypothesis will be rejected if max{*T*^(1)^,…,*T*^(*d*)^}>*z*_1−*α*,1_(**R**) or if $\max \{{CI}_{MCP,l}^{(1)},\ldots, {CI}_{MCP,l}^{(d)}\} > {AUC}_{0}$. The correlation matrix **R**, however, is unknown and must be replaced by a consistent estimator $\widehat {\mathbf {R}}$. We propose to replace **R** by $\widehat {\mathbf {R}}$ in the considerations above, where $\widehat {\mathbf {R}} = [\widehat {r}^{(\ell,m)}]_{\ell,m=1,\ldots,d}$ and $\widehat {r}^{(\ell,m)}= \tfrac {\widehat {v}^{(\ell,m)}}{\sqrt {\widehat {v}^{(\ell,\ell)} \widehat {v}^{(m,m)}}}$, respectively.

Simulation studies indicate, however, that the speed of convergence of **T** to a multivariate normal distribution is quite slow, particularly when smaller sample sizes and larger numbers of diagnostic tests are considered. In a variety of applications, see e.g. Zou and Yue [28] or Konietschke et al. [10], it turns out that the use of adequate transformations (e.g., the Logit-transformation) tend to increase the speed of convergence. Therefore, simultaneous confidence intervals with Logit transformation will be derived in the next section.

#### Multiple contrast tests and simultaneous confidence intervals with Logit transformation

To derive simultaneous Logit-transformed confidence intervals let

$$\begin{array}{@{}rcl@{}} \mathbf{g}(\mathbf{AUC}) &= \left(g(AUC^{(1)}),\ldots, g(AUC^{(d)})\right): (0,1)^{d} \to \mathbb{R}^{d}, \end{array} $$

denote the vector of Logit-transformed AUC’s, where

$$\begin{array}{@{}rcl@{}} g(AUC^{(\ell)}) &= \log\left(\frac{AUC^{(\ell)}}{1-AUC^{(\ell)}} \right). \end{array} $$

Furthermore, let

$${} {\small{\begin{aligned} \boldsymbol{\Psi}=diag\left(\frac{1}{AUC^{(1)}(1-AUC^{(1)})},\ldots, \frac{1}{AUC^{(d)}(1-AUC^{(d)})}\right) \end{aligned}}} $$

denote the diagonal Jacobian matrix of **g**(**AUC**). Under the additional assumption that *N*→*∞* such that *N*/*n*_*i*_→*f*_*i*_, it follows from Cramer’s multivariate *δ*-theorem (see, e.g., Ferguson [29], Theorem 7.4) that

(14) $$\begin{array}{@{}rcl@{}} \sqrt{N} \left(\mathbf{g} \left(\widehat{\mathbf{AUC}}\right) - \mathbf{g}\left(\mathbf{AUC} \right) \right) \stackrel{\mathcal{D}}{\to} N \left(\mathbf{0}, \mathbf{S}_{N}\right) \end{array} $$

where **S**_*N*_=***Ψ*V**_*N*_***Ψ***^′^ and **V**_*N*_ is given in (6). To estimate the asymptotic covariance matrix **S**_*N*_, let

$${} {\small{\begin{aligned} \widehat{\boldsymbol{\Psi}}= diag\left(\frac{1}{\widehat{AUC}^{(1)}(1-\widehat{AUC}^{(1)})},\ldots, \frac{1}{\widehat{AUC}^{(d)}(1-\widehat{AUC}^{(d)})}\right) \end{aligned}}} $$

denote the estimated Jacobian matrix of **g**(**AUC**) and note that the estimator $\widehat {\mathbf {S}}_{N} = \widehat {\boldsymbol {\Psi }} \widehat {\mathbf {V}}_{N} \widehat {\boldsymbol {\Psi }}$ is a consistent estimator of **S**_*N*_. Again there is a problem if $\widehat {AUC}^{(\ell)}=0$ or 1. Here, $\widehat {\boldsymbol {\Psi }}$ and in turn $\widehat {\mathbf {S}}_{N}$ cannot be calculated. This problem is addressed in the analysis of the example in the Results section. To test the individual hypothesis $H_{0}^{(\ell)}: AUC^{(\ell)} \leq {AUC}_{0}$ define the pivotal quantities

(15) $$ \begin{aligned} \widetilde{T}^{(\ell)} &= \left(g(\widehat{AUC}^{(\ell)}) - g(AUC^{(\ell)})\right)\sqrt{\frac{N}{\widehat{s}^{(\ell,\ell)}}} \stackrel{\mathcal{D}}{\to} N(0,1),\\&\quad \; N \to \infty, \; \ell=1,\ldots,d, \end{aligned}  $$

where $\widehat {s}^{(\ell,\ell)}$ denotes the *ℓ*-th diagonal element of $\widehat {\mathbf {S}}_{N}.$ The joint distribution of the vector $\widetilde {\mathbf {T}}=(\widetilde {T}^{(1)},\ldots, \widetilde {T}^{(d)})'$ is given in the next theorem.

##### **Theorem2**.

If *N*→*∞* such that *N*/*n*_*i*_→*f*_*i*_<*∞*, then the vector $\widetilde {\mathbf {T}}\,=\,(\widetilde {T}^{(1)},\ldots, \widetilde {T}^{(d)})'$ follows, asymptotically, a multivariate normal distribution with expectation **0** and correlation matrix **R**, where **R** is given in Theorem 1.

It follows from Theorem 2 that both the vectors **T** and $\widetilde {\mathbf {T}}$ have, asymptotically, as *N*→*∞*, the same joint distribution. Both the correlation matrices of **T** and $\widetilde {\mathbf {T}}$ asymptotically coincide due to the diagonal structure of ***Ψ***. Now, a simultaneous test procedure, which takes the correlation into account can be derived. The individual null hypothesis $H_{0}^{(\ell)}: AUC^{(\ell)} \leq {AUC}_{0}$ will be rejected at multiple level *α*, if

(16) $$\begin{array}{@{}rcl@{}} \widetilde{T}^{(\ell)} \geq z_{1-\alpha,1}(\widehat{\mathbf{R}}), \end{array} $$

where $z_{1-\alpha,1}(\widehat {\mathbf {R}})$ denotes the one-sided equicoordinate quantile of the corresponding multivariate normal distribution where the correlation matrix **R** is replaced with the consistent estimator $\widehat {\mathbf {R}}$. One-sided simultaneous confidence intervals for *AUC*^(*ℓ*)^ are then given by

(17) $$ {\small{\begin{aligned} {CI}_{Logit}^{(\ell)} = \left[expit\left(g\left(\widehat{AUC}^{(\ell)}\right) - z_{1-\alpha,1}(\widehat{\mathbf{R}})\sqrt{\frac{\widehat{s}^{(\ell,\ell)}}{N}}\right),1 \right], \end{aligned}}}  $$

where $expit(y) = \tfrac {exp(y)}{1+\exp (y)}$ denotes the inverse Logit-transformation. The global null hypothesis *H*_0_:**AUC**≤*AUC*_0_·**1** will be rejected, if $\max \left \{\widetilde {T}^{(1)},\ldots,\widetilde {T}^{(d)}\right \} \geq z_{1-\alpha,1}(\widehat {\mathbf {R}})$, or if $\max \{{CI}_{Logit,l}^{(1)},\ldots, {CI}_{Logit,l}^{(d)}\} > {AUC}_{0}$. Since the Logit-function is monotone, the procedure asymptotically controls the familywise error rate in the strong sense [26].

### Small sample approximations with Wild Bootstrap

In the previous section approaches for the selection of diagnostic tests based on the AUC’s have been derived. The procedures are based on the asymptotic joint distribution of the vectors **T** or $\widetilde {\mathbf {T}}$, respectively. The proposed approaches for selection of diagnostic tests are valid for large sample sizes. In order to investigate the accuracies of the procedures in terms of (i) controlling the pre-assigned type-I error level under the null hypothesis, (ii) maintaining the nominal coverage probability of the corresponding simultaneous confidence intervals, and (iii) their powers to detect certain alternatives, extensive simulation studies were conducted.

These simulation studies indicate, however, that both the statistics **T** in (12) and $\widetilde {\mathbf {T}}$ in (15) tend to result in liberal or conservative decisions in case of smaller sample sizes (*N*≤100) and larger AUC (*AUC*≥0.8). The results are in concordance with the simulation results proposed for univariate statistics by Kottas et al. [15] or Qin and Hotilovac [30]. Therefore, we propose a Wild Bootstrap approach to approximate their sampling distributions for small sample sizes.

Resampling procedures are widely known to be quite robust methods, even for small sample sizes. However, permutation methods cannot be used in this setup, since the distributions of the test statistics and the resampling statistics do not coincide, not even asymptotically (Pauly M, Asendorf T, Konietschke F: Permutation tests and confidence intervals for the area under the ROC curve, submitted). Simulation studies indicate that the use of the conventional Bootstrap from Efron [31] results in liberal conclusions, particularly when confronted with an *AUC*≥0.7 (see Table 1). Therefore, we did not further investigate the conventional Bootstrap. In contrast, the Wild Bootstrap approach ensures that the resampling distribution of the statistics mimics the distribution of **T** and $\widetilde {\mathbf {T}}$, asymptotically. The Wild Bootstrap technique is motivated by the residual bootstrap commonly applied in regression analysis [32-35], and in time-series testing problems [36-38]. It is also proposed in the context of survival analysis [39-42], and will be explained in the following.

**Table 1:** **Empirical type-I error (theoretical** ***2.5%*** **) of the normal Bootstrap for** ***d=5*** **and** ***N=50*** **with varying case-control-ratio and varying AUC**

**ccr**	***AUC***				
	**0.5**	**0.6**	**0.7**	**0.8**	**0.9**
1:1	1.68*%*	2.28*%*	3.10*%*	5.00*%*	7.80*%*
1:4	1.90*%*	2.96*%*	4.70*%*	6.40*%*	12.10*%*

Let

(18) $$\begin{array}{@{}rcl@{}}  \left(W_{01},\ldots,W_{0n_{0}}, W_{11}, \ldots, W_{1n_{1}}\right) \end{array} $$

denote independent and identically distributed random weights with *E*(*W*_*is*_)=0 and *Var*(*W*_*is*_)=1, which are independent of the data. We will investigate three different kinds of random weights *W*_*is*_ in our extensive simulation study:

- Rademacher weights: $P(W_{\textit {is}}=1) = P(W_{\textit {is}}=-1)=\frac 12$.
- Standard normal weights: $\phantom {\dot {i}\!}W_{01},\ldots,W_{1n_{1}} \sim N(0,1)$.
- Uniform weights: $W_{01},\ldots, W_{1n_{1}} \sim U\left [-\frac {\sqrt {12}}{2}, \frac {\sqrt {12}}{2}\right ]$.

Let

(19) $$ {} {\small{\begin{aligned} \mathbf{Z}^{\ast}_{is} &=W_{is}\cdot\left(\mathbf{Z}_{is}- \overline{\mathbf{Z}}_{i\cdot}\right) \\ &= \left(W_{is}\cdot\left(Z_{is}^{(1)} - \overline{Z}_{i\cdot}^{(1)}\right),\ldots, W_{is}\cdot\left(Z_{is}^{(d)} - \overline{Z}_{i\cdot}^{(d)}\right)\right),\\ &\qquad i=0,1, \; s=1,\ldots, n_{i}, \end{aligned}}}  $$

denote *N* resampling vectors, where **Z**_*is*_ is given in (8). Furthermore, let $\overline {\mathbf {Z}}^{\ast }_{i\cdot } = n_{i}^{-1}\sum _{k=1}^{n_{i}}\mathbf {Z}^{\ast }_{is} = \left (\overline {Z}_{i\cdot }^{\ast (1)},\ldots, \overline {Z}_{i\cdot }^{\ast (d)} \right)' $ denote their means and let

$$\begin{array}{@{}rcl@{}} \widehat{v}_{i}^{\,\ast(\ell,\ell)} = \frac{1}{n_{i}-1} \sum_{s=1}^{n_{i}} \left(Z_{is}^{\ast(\ell)} - \overline{Z}_{i\cdot}^{\ast(\ell)} \right)^{2} \end{array} $$

denote the empirical variance of $Z_{i1}^{\ast (\ell)},\ldots, Z_{{in}_{i}}^{\ast (\ell)}$, *ℓ*=1,…,*d*. In the next theorem it will be shown that the conditional resampling distribution of the vector

(20) $$  \begin{aligned} \mathbf{T}^{\ast} &= \left(T^{\ast(1)},\ldots, T^{\ast(d)} \right)', \; \text{where}\\ \; T^{\ast(\ell)} &= \sqrt{N} \frac{\overline{Z}_{1\cdot}^{\ast(\ell)} - \overline{Z}_{0\cdot}^{\ast(\ell)}}{\sqrt{\widehat{v}_{1}^{\,\ast(\ell,\ell)}/n_{0} + \widehat{v}_{0}^{\,\ast(\ell,\ell)}/n_{1}}}, \end{aligned}  $$

mimics the distribution of both the vectors **T** and $\widetilde {\mathbf {T}}$, asymptotically.

#### **Theorem3**.

If *N*→*∞* such that $\tfrac {N}{n_{i}}$ converges to some finite constant *f*_*i*_, then the conditional distribution of **T**^∗^ given the data **X** converges in probability to the multivariate normal distribution with expectation **0** and correlation matrix **R**.

For proof see Additional file 1. Note that Theorem 3 is valid under the null as well as under the alternative, i.e., the resampling distribution mimics the distributions of **T** and $\widetilde {\mathbf {T}}$ for arbitrary values of **AUC**=(*AUC*^(1)^,…,*AUC*^(*d*)^)^′^. Next we will explain the computation of the simultaneous confidence intervals:

- Given the data **X**, compute the point estimators $\widehat {\mathbf {AUC}}$ and $\widehat {\mathbf {V}}_{N}$ as given in (5) and (9), respectively.
- Generate *N*=*n*_0_+*n*_1_ random weights $W_{01},\ldots, W_{1n_{1}}\phantom {\dot {i}\!}$ as described in (18)
- Compute $A^{\ast }_{j}:=\max \{T^{\ast (1)},\ldots,T^{\ast (d)}\}$ as given in (20).
- Repeat the steps 2. - 3. *nboot* times (e.g. *nboot*=10,000) and obtain the values $A_{1}^{\ast },\ldots, A_{\textit {nboot}}^{\ast }$.
- Compare each $A_{j}^{\ast }$ with $\max \left \{\widetilde {\mathbf {T}}\right \}$. Then the individual p-value for $H_{0}^{(\ell)}:AUC^{(\ell)}\leq {AUC}_{0}$ is obtained from $\tfrac {1}{nboot}\sum _{j=1}^{nboot}\mathcal {I}\{\widetilde {T}^{(\ell)}\geq A^{\ast }_{j}\}$, where $\mathcal {I}\{\cdot \}$ denotes the indicator function.
- Estimate the quantile *z*_1−*α*,1_(**R**) by the one-sided (1−*α*)-quantile $z^{\ast }_{1-\alpha,1}$ of $A_{1}^{\ast },\ldots, A_{\textit {nboot}}^{\ast }$ to obtain the one-sided (1−*α*) simultaneous confidence intervals given by

(21) $${}</p><p class="noindent">{\small{\begin{aligned} {CI}_{WB}^{\ast(\ell)} = \left[expit\left(g\left(\widehat{AUC}^{(\ell)}\right) - z^{\ast}_{1-\alpha,1}\sqrt{\frac{\widehat{s}^{(\ell,\ell)}}{N}}\right),1 \right]. \end{aligned}}}  $$

## Results

### Simulation results

We performed a simulation study to investigate the properties of the different approaches. All simulations were conducted with R environment, version 2.15.2. (R Development Core Team, 2010), each with 5, 000 simulation runs and 5, 000 bootstrap repetitions. The nominal type-I error was set to 2.5*%* one-sided and the global null hypothesis according to (4) was rejected, if at least one of the one-sided p-values was smaller than *α*=2.5*%*. This means, the family wise error rate in the strong sense (FWER) is controlled, and the one-sided empirical type-I error should be closed to 2.5*%*. It is also possible to use the corresponding confidence intervals for decision. Then the global null hypothesis is rejected if the lower limit of at least one confidence interval was above *AUC*_0_.

We generated multivariate normally distributed random vectors with compound symmetric correlation structure and defined the following scenario as standard scenario: a total sample size *N*=100 with a case-control ratio (ccr) of 1:1, *d*=5 diagnostic tests and a correlation of *ρ*=0.9 between the tests (motivated by [2,13,24]; and the example data set). The different parameters and conditions were varied afterwards as follows:

- The true *AUC* (0.5,…, 0.9)
- The number of diagnostic tests *d* (5, 10, 20)
- The total sample size *N* (50, 100, 200)
- The case-control ratio *ccr* (1:1, 1:2, 1:4, 1:9)
- The true correlation between the diagnostic tests *ρ* (0.3, 0.6, 0.9)
- The covariance structure in the data (compound symmetry, unstructured, and diagonal matrix with heterogeneous variances and positive or negative pairing)
- The distribution of the data (normal, skewed = log-normal, ordinal)

The different parameter constellations and all simulation results can be seen in the Additional file 2. Due to computational complexity, and its weak behavior in standard situations, we did not further investigate the conventional Bootstrap in our simulation study.

In a first step, this standard scenario was used for the comparison of the three random weights for the Wild Bootstrap: Rademacher (WB-Rade), standard normal (WB-Normal) and uniform (WB-Unif) weights. The results are displayed in the Additional file 3. For an AUC of 0.5 the three weights lead to nearly the same empirical type-I error and are quite conservative (empirical *α*≈0.015). For larger AUC’s the results are less conservative and for AUC’s above 0.8 the empirical type-I error is around 2.5*%*. The Wild Bootstrap approach with uniform weights is, however, more conservative, while the standard normal and the Rademacher weights lead nearly to the same results. Therefore, and to present the simulation results more clearly, we only consider the standard normal weights in the following. The simulation results for the other weights are provided in the Additional file 2.

**Figure 3:**
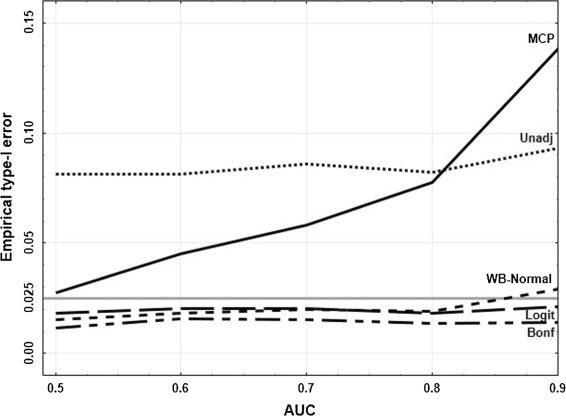
Empirical type-I error for varying AUC’s. Empirical type-I error of the different approaches for the standard scenario (see text) with varying AUC’s.

In practice often unadjusted (with the local type-I error *α*_0_ equal to the global type-I error *α*) or Bonferroni adjusted confidence intervals for the single AUC’s are used (see for example Shiotani et al. [43]). Therefore, in a second step, we compared these approaches (again for the standard scenario) using the multiple contrast test (‘MCP’), the simultaneous Logit (‘Logit’) and the Wild Bootstrap (‘WB-Normal’) approach. In Figure 3 it becomes apparent that unadjusted intervals (‘Unadj’) lead to highly liberal conclusions (empirical type-I error 8−9*%*), while the Bonferroni correction (‘Bonf’) is too conservative (1.1−1.5*%*). Therefore we will not consider these approaches in the sequel. The MCP approach keeps the type-I error for an AUC of 0.5, but becomes more and more liberal for larger AUC’s (up to 14*%* for *AUC*=0.9). The empirical type I error of the Logit and the WB-Normal approach is comparable and between 1.5*%* and 2.9*%*. In the following we will investigate the influence of the different parameter settings on the type-I error of the Logit and the WB-Normal approach, and also of the MCP approach as the basis of both the approaches (despite of its liberal behavior).

The strength of the correlation, the type of the covariance structure and a skewed distribution do not have any impact on the behavior of the test (see figures and tables in the Additional files 2, 4, 5, and 6).

**Figure 4:**
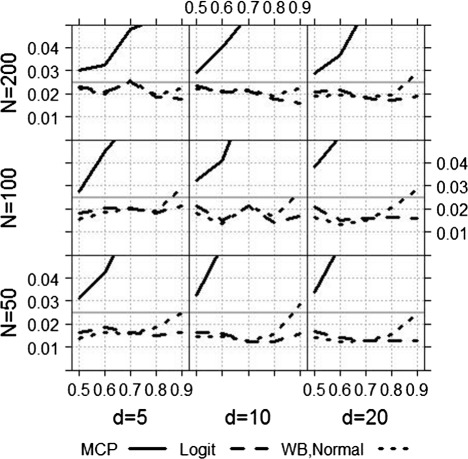
Empirical type-I error for varying *N* and *d*. Empirical type-I error of the MCP, the Logit and the WB-Normal approach for varying sample size and number of diagnostic tests.

The impact of the sample size *N* and the number of diagnostic tests *d* is shown in Figure 4. As expected, for a larger sample size and a small number of diagnostic tests the type-I error is better exploited. As already seen in Figure 3 the Logit and the WB-Normal approach are comparable if *AUC*≤0.8 (independent of *N* and *d*). For larger AUC’s, the WB-Normal approach leads to a larger empirical type-I error. On the one hand, this means that *α* is better exploited, on the other hand, this means that the results are liberal. The empirical type-I error of the Logit approach for *AUC*=0.9 ranges from 1.3*%* to 2.1*%*, and of the WB-Normal approach from 2.2*%* to 2.9*%*.

**Figure 5:**
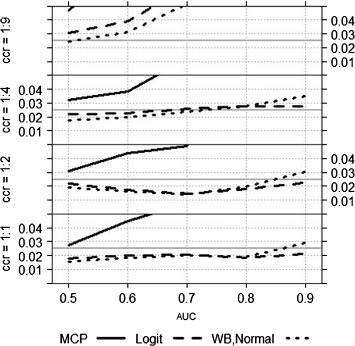
Empirical type-I error for varying ccr. Empirical type-I error of the MCP, the Logit and the WB-Normal approach for varying case-control ratios.

If the case-control ratio (ccr) is not balanced, the empirical type-I error increases with increasing imbalance (see Figure 5). For an AUC of 0.8 or smaller both approaches are robust to an imbalance up to 1:4. For *AUC*=0.9 the liberality of the WB-Normal approach is a disadvantage here, the empirical type-I error is above 2.5*%*. For a case-control ratio of 1:9, both approaches are far too liberal.

Ordinal data was generated using discretised normal distributions with a given AUC. For this data, representing a 5-point grading scale, the empirical type-I error decreases with increasing AUC (*AUC*=0.5: Logit = 2.3*%*, WB-Normal = 2.2*%* to *AUC*=0.9: Logit = 1.7*%*, WB-Normal = 1.6*%*). For details see Additional file 2.

The power was calculated for one example scenario (*N*=200, *d*=5, *ccr*=1:1, *ρ*=0.9, *AUC*_0_=0.7), where the empirical type-I error of the Logit and of the WB-Normal approach was nearly the same. The true AUC is increasing from 0.7 (which is equal to *AUC*_0_) to 0.85, according *ΔAUC*=0,…,0.15. The power of the two approaches is basically the same. For an *ΔAUC* of 0.1 (i.e. *AUC*=0.8 vs. *AUC*_0_=0.7) the power is greater than 80*%* (see Additional file 2).

### Results for the analysis of the example

**Figure 6:**
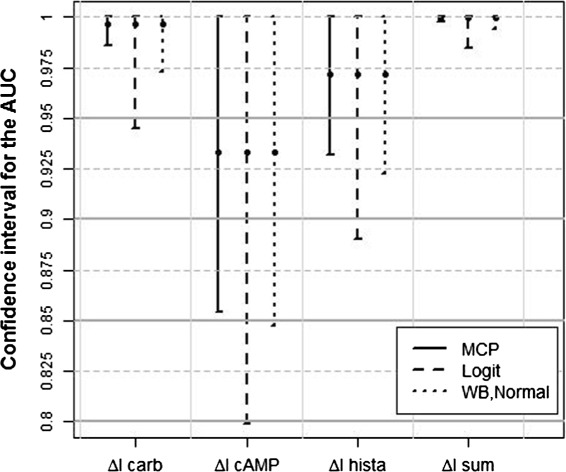
Confidence intervals for the biomarkers. One-sided 97.5*%* confidence intervals for the four biomarkers using the MCP, the Logit, and the WB-Normal approach.

The point estimators for the AUC’s are presented in the Background section in Figure 2. The number of 26 cases and 41 controls correspond to a case-control ratio of 1:1.6. The Spearman correlation coefficients between the biomarkers range from 0.64 to 0.95. For *ΔI*_*sum*_ the result was AUC=1. Because *logit*(1)=*∞*, we modified the data for *ΔI*_*sum*_ such that we replaced the largest measurement of the controls with the smallest measurement of the cases. This minimal change leads to a point estimator for the AUC of 0.9999, and enables us to calculate the confidence intervals. This replacement strategy is conservative, since the effect is decreased, and the variance is increased. The one-sided 97.5*%* confidence intervals for all biomarkers using the MCP, the Logit, and the Wild Bootstrap approach are displayed in Figure 6. The results of the Wild Bootstrap with the three different weights differed just in the third decimal place. For consistency we displayed the WB-Normal approach here. The pattern of the results is the same for all four biomarkers. According to the simulation results, the MCP intervals are the shortest, the Logit intervals are the broadest, and the WB intervals are in between.

In the article of Derichs et al. [3] no threshold is defined. In Figure 6 four possible thresholds (0.8,0.85,0.9,0.95) are marked by solid horizontal lines. In Table 2 for each of these thresholds the numbers of selected biomarkers, depending on the individual approach, are listed. Apparently, the Logit approach is a more conservative selection criterion than the Wild Bootstrap approach. Although the MCP intervals are clearly shorter than the Wild Bootstrap intervals, the number of selected biomarkers is the same for the MCP and the WB approach for three thresholds. Only for the threshold of 0.85 the MCP approach would select one biomarker more. Considering the simulation results of this section we would recommend to use the WB-Normal approach.

**Table 2:** **Number of selected biomarkers of the MCP, the Logit, and the WB-Normal approach for different thresholds (based on one-sided** ***97.5%*** **confidence intervals)**

**Threshold**	**MCP**	**Logit**	**WB-Normal**
0.8	4	3	4
0.85	4	3	3
0.9	3	2	3
0.95	2	1	2

## Discussion

It is widely discussed in the literature, whether the type-I error should be adjusted for multiplicity and whether the Bonferroni correction is an appropriate approach. Among many others, Wittes [44] states that lack of adjustment can lead to a misinterpretation of the study results as well as Bonferroni adjustment can do. Furthermore Perneger [45] states that “In summary, Bonferroni adjustments have, at best, limited applications in biomedical research, and should not be used when assessing evidence about specific hypotheses”. Nevertheless, in practice often Bonferroni adjusted or even unadjusted confidence intervals for the single AUC’s are used (see for example [43]). Konietschke et al. [10] proposed nonparametric multiple contrast tests and simultaneous confidence intervals for adequate correction of the type-I error, which take the dependencies within the data into account. Furthermore the authors recommended the transformation method (for example the Logit-transformation) to get less liberal results. However, Qin and Hotilovac [30] noticed that the Logit-transformed intervals are conservative for high accuracies. The reason is that the estimator $logit(\widehat {AUC})$ is quite unstable if $\widehat {AUC}$ is close to 0 or 1 because of a possibly larger variance. Obuchowski and Lieber [46] compared different confidence intervals for the AUC and concluded that for small sample sizes none of them provides adequate coverage for high accuracies.

## Conclusion

In this article we derived a Wild Bootstrap approach, which exploits the type-I error much better than the Logit-approach, even for high accuracies and small samples. Neither the strength of correlation, nor the structure of the covariance matrix, nor a skewed distribution, nor a moderate imbalanced case-control ratio has any impact on this desirable property of the Wild Bootstrap approach. Corresponding to these results we recommend to use the Wild Bootstrap approach with standard normally distributed weights for the selection of biomarkers in early diagnostic trials with the AUC as selection criterion.

## Additional files

Additional file 1
**Proof of Theorem 3.**

Additional file 2
**Tables of simulation results.**

Additional file 3
**Figure S1.** Empirical type-I error of the Wild Bootstrap approach with the three different weights for the standard scenario (see article, Section “Simulation results”) with varying AUC’s.

Additional file 4
**Figure S2.** Empirical type-I error of the MCP, the Logit and the WB-Normal approach for varying strength of correlation.

Additional file 5
**Figure S3.** Empirical type-I error of the MCP, the Logit and the WB-Normal approach for different covariance structures (CS: compound symmetry, UN: unstructured, PP/NP: diagonal matrix with heterogeneous variances and positive/negative pairing).

Additional file 6
**Figure S4.** Empirical type-I error of the MCP, the Logit and the WB-Normal approach for normal and log-normal distributed data.
