# Supplementary material for: A Wild Bootstrap approach for the selection of biomarkers in early diagnostic trials
Source: BMC Med Res Methodol. 2015 May 1;15:43. doi: 10.1186/s12874-015-0025-y (PMC4426186; doi:10.1186/s12874-015-0025-y)
Supplement: Additional file 1 — Proof of Theorem 3. [file 12874_2015_25_MOESM1_ESM.pdf]

Additional file 1 — Proof of Theorem 3

Note that

$$\mathbf{A}_{is} = \frac{1}{\sqrt{n_i}} \mathbf{Z}^*_{is}, \ i = 0, 1; \ k = 1, \ldots, n_i,$$

defines an array of row-wise independent random vectors given the data $\mathbf{X}$. It fulfils

$$E\left(\mathbf{A}_{is} \middle| \mathbf{X}\right) = \mathbf{0} \quad and \quad Var\left(\mathbf{A}_{is} \middle| \mathbf{X}\right) = \frac{1}{n_i} \left(\mathbf{Z}_{is} - \overline{\mathbf{Z}}_{i.}\right)\left(\mathbf{Z}_{is} - \overline{\mathbf{Z}}_{i.}\right)'.$$

Hence, we have

$$\begin{aligned}
& E\left(\sqrt{N}\left(\overline{\mathbf{Z}}^*_{0\cdot} - \overline{\mathbf{Z}}^*_{1\cdot}\right) \middle| \mathbf{X}\right) = \mathbf{0} \\
& Var\left(\sqrt{N}\left(\overline{\mathbf{Z}}^*_{0\cdot} - \overline{\mathbf{Z}}^*_{1\cdot}\right) \middle| \mathbf{X}\right) \\
= \ & N\left(\frac{1}{n_0^2}\sum_{s=1}^{n_0}\left(\mathbf{Z}_{0s} - \overline{\mathbf{Z}}_{0.}\right)\left(\mathbf{Z}_{0s} - \overline{\mathbf{Z}}_{0.}\right)' + \frac{1}{n_1^2}\sum_{s=1}^{n_1}\left(\mathbf{Z}_{1s} - \overline{\mathbf{Z}}_{1.}\right)\left(\mathbf{Z}_{1s} - \overline{\mathbf{Z}}_{1.}\right)'\right) \\
= \ & \frac{N}{n_0}\frac{1}{n_0}\sum_{s=1}^{n_0}\left(\mathbf{Z}_{0s} - \overline{\mathbf{Z}}_{0.}\right)\left(\mathbf{Z}_{0s} - \overline{\mathbf{Z}}_{0.}\right)' + \frac{N}{n_1}\frac{1}{n_1}\sum_{s=1}^{n_1}\left(\mathbf{Z}_{1s} - \overline{\mathbf{Z}}_{1.}\right)\left(\mathbf{Z}_{1s} - \overline{\mathbf{Z}}_{1.}\right)' \xrightarrow{P} \mathbf{V}_N.
\end{aligned}$$

It follows with the same arguments as in Lemma 1 in [1], or Theorem 2.1 in [2], that the conditional distribution of $\sqrt{N}(\overline{\mathbf{Z}}^*_{0\cdot} - \overline{\mathbf{Z}}^*_{1\cdot})$ given the observed data $\mathbf{X}$ has, asymptotically, a multivariate normal distribution with expectation $\mathbf{0}$ and covariance matrix $\mathbf{V}_N$. The conditional consistency of the variances $\widehat{v}_i^{*(\ell,\ell)}$ follows with the same arguments as in Theorem 1 in [3], and the result follows from Slutzky's theorem.

References

[1] Pauly M. and Brunner E. and Konietschke F. 2014. Asymptotic permutation tests in general factorial designs. *Journal of the Royal Statistical Society - Series B.* In Press, DOI: 10.1111/rssb.12073.

[2] Pauly M. 2011. Weighted resampling of martingale difference arrays with applications. *Electronic Journal of Statistics* **5,** 41–42.

[3] Konietschke F. and Pauly M. 2014. Bootstrapping and permuting paired t-test type statistics. *Statistics and Computing* **24,** 283 – 296.
