# Supplementary material for: A Wild Bootstrap approach for the selection of biomarkers in early diagnostic trials
Source: BMC Med Res Methodol. 2015 May 1;15:43. doi: 10.1186/s12874-015-0025-y (PMC4426186; doi:10.1186/s12874-015-0025-y)
Supplement: Additional file 2 — Tables of simulation results. [file 12874_2015_25_MOESM2_ESM.pdf]

Tables of simulation results

Table 1: The results of the standard scenario ($N = 100$, $d = 5$, $ccr = 1:1$, $\rho = 0.9$) with varying AUC's for the comparison of the three Wild Bootstrap weights (Fig. S1) and for the comparison of all other approaches (Fig. 3).

AUC	Unadj	Bonf	MCP	Logit	WB,Rade	WB,Normal	WB,Unif
0.5	0.0814	0.0116	0.0274	0.0180	0.0160	0.0154	0.0152
0.6	0.0814	0.0158	0.0450	0.0202	0.0174	0.0184	0.0182
0.7	0.0860	0.0152	0.0580	0.0204	0.0176	0.0200	0.0194
0.8	0.0822	0.0136	0.0774	0.0182	0.0170	0.0192	0.0192
0.9	0.0930	0.0140	0.1380	0.0212	0.0250	0.0290	0.0276

Table 2: The results of the standard scenario ($N = 100$, $d = 5$, $ccr = 1:1$) with varying AUC's and varying strength of correlation for the comparison of the different approaches (Fig. S2).

rho	AUC	MCP	Logit	WB,Rade	WB,Normal	WB,Unif
0.3	0.5	0.0326	0.0208	0.0168	0.0188	0.0172
0.3	0.6	0.0470	0.0218	0.0184	0.0208	0.0198
0.3	0.7	0.0630	0.0210	0.0178	0.0210	0.0188
0.3	0.8	0.0766	0.0146	0.0134	0.0162	0.0150
0.3	0.9	0.1438	0.0184	0.0200	0.0264	0.0244
0.6	0.5	0.0284	0.0174	0.0148	0.0164	0.0152
0.6	0.6	0.0420	0.0198	0.0172	0.0184	0.0182
0.6	0.7	0.0548	0.0190	0.0174	0.0184	0.0178
0.6	0.8	0.0804	0.0202	0.0196	0.0230	0.0210
0.6	0.9	0.1310	0.0158	0.0182	0.0226	0.0218
0.9	0.5	0.0274	0.0180	0.0160	0.0154	0.0152
0.9	0.6	0.0450	0.0202	0.0174	0.0184	0.0182
0.9	0.7	0.0580	0.0204	0.0176	0.0200	0.0194
0.9	0.8	0.0774	0.0182	0.0170	0.0192	0.0192
0.9	0.9	0.1380	0.0212	0.0250	0.0290	0.0276

Table 3: The results of the standard scenario ($N = 100$, $d = 5$, $ccr = 1 : 1$, $\rho = 0.9$) with varying AUC's and different covariance structures for the comparison of the different approaches (Fig. S3).

covariance structure	AUC	MCP	Logit	WB,Rade	WB,Normal	WB,Unif
CS	0.5	0.0274	0.0180	0.0160	0.0154	0.0152
CS	0.6	0.0450	0.0202	0.0174	0.0184	0.0182
CS	0.7	0.0580	0.0204	0.0176	0.0200	0.0194
CS	0.8	0.0774	0.0182	0.0170	0.0192	0.0192
CS	0.9	0.1380	0.0212	0.0250	0.0290	0.0276
US	0.5	0.0310	0.0188	0.0156	0.0170	0.0172
US	0.6	0.0452	0.0210	0.0166	0.0192	0.0184
US	0.7	0.0616	0.0180	0.0162	0.0194	0.0172
US	0.8	0.0930	0.0182	0.0162	0.0204	0.0180
US	0.9	0.1374	0.0248	0.0294	0.0350	0.0314
PP	0.5	0.0364	0.0228	0.0152	0.0188	0.0174
PP	0.6	0.0448	0.0182	0.0138	0.0164	0.0150
PP	0.7	0.0604	0.0206	0.0164	0.0200	0.0182
PP	0.8	0.0958	0.0174	0.0158	0.0186	0.0170
PP	0.9	0.1624	0.0202	0.0226	0.0288	0.0266
NP	0.5	0.0262	0.0184	0.0164	0.0172	0.0164
NP	0.6	0.0420	0.0174	0.0140	0.0156	0.0144
NP	0.7	0.0598	0.0170	0.0126	0.0166	0.0148
NP	0.8	0.0896	0.0178	0.0152	0.0206	0.0180
NP	0.9	0.1604	0.0192	0.0214	0.0284	0.0240

Table 4: The results of the standard scenario ($N = 100$, $d = 5$, $ccr = 1 : 1$, $\rho = 0.9$) with varying AUC's and for normal and log-normal distributed data for the comparison of the different approaches (Fig. S4).

distribution	AUC	MCP	Logit	WB,Rade	WB,Normal	WB,Unif
normal	0.5	0.0274	0.0180	0.0160	0.0154	0.0152
normal	0.6	0.0450	0.0202	0.0174	0.0184	0.0182
normal	0.7	0.0580	0.0204	0.0176	0.0200	0.0194
normal	0.8	0.0774	0.0182	0.0170	0.0192	0.0192
normal	0.9	0.1380	0.0212	0.0250	0.0290	0.0276
log-normal	0.5	0.0300	0.0202	0.0168	0.0184	0.0166
log-normal	0.6	0.0460	0.0198	0.0166	0.0196	0.0174
log-normal	0.7	0.0596	0.0182	0.0154	0.0178	0.0166
log-normal	0.8	0.0790	0.0160	0.0154	0.0178	0.0164
log-normal	0.9	0.1250	0.0150	0.0174	0.0216	0.0206

Table 5: The results for varying d, N and AUC's for the comparison of the different approaches (Fig. 4).

d	N	AUC	MCP	Logit	WB,Rade	WB,Normal	WB,Unif
5	50	0.5	0.0310	0.0164	0.0110	0.0136	0.0128
5	50	0.6	0.0422	0.0184	0.0150	0.0166	0.0168
5	50	0.7	0.0648	0.0162	0.0128	0.0160	0.0158
5	50	0.8	0.0866	0.0150	0.0154	0.0184	0.0174
5	50	0.9	0.1404	0.0162	0.0234	0.0248	0.0244
5	100	0.5	0.0274	0.0180	0.0160	0.0154	0.0152
5	100	0.6	0.0450	0.0202	0.0174	0.0184	0.0182
5	100	0.7	0.0580	0.0204	0.0176	0.0200	0.0194
5	100	0.8	0.0774	0.0182	0.0170	0.0192	0.0192
5	100	0.9	0.1380	0.0212	0.0250	0.0290	0.0276
5	200	0.5	0.0302	0.0232	0.0222	0.0226	0.0216
5	200	0.6	0.0324	0.0206	0.0192	0.0198	0.0198
5	200	0.7	0.0478	0.0254	0.0240	0.0256	0.0250
5	200	0.8	0.0524	0.0184	0.0178	0.0196	0.0180
5	200	0.9	0.0858	0.0178	0.0186	0.0222	0.0210
10	50	0.5	0.0324	0.0166	0.0114	0.0146	0.0128
10	50	0.6	0.0540	0.0160	0.0124	0.0146	0.0138
10	50	0.7	0.0612	0.0124	0.0092	0.0126	0.0114
10	50	0.8	0.0966	0.0122	0.0120	0.0156	0.0144
10	50	0.9	0.1806	0.0160	0.0276	0.0286	0.0272
10	100	0.5	0.0326	0.0212	0.0160	0.0182	0.0192
10	100	0.6	0.0408	0.0152	0.0116	0.0136	0.0124
10	100	0.7	0.0698	0.0212	0.0158	0.0212	0.0196
10	100	0.8	0.0958	0.0140	0.0122	0.0168	0.0154
10	100	0.9	0.1704	0.0170	0.0206	0.0272	0.0250
10	200	0.5	0.0292	0.0234	0.0206	0.0222	0.0206
10	200	0.6	0.0400	0.0208	0.0196	0.0214	0.0198
10	200	0.7	0.0542	0.0212	0.0198	0.0216	0.0206
10	200	0.8	0.0596	0.0178	0.0164	0.0188	0.0172
10	200	0.9	0.1114	0.0160	0.0168	0.0226	0.0204
20	50	0.5	0.0340	0.0170	0.0120	0.0142	0.0134
20	50	0.6	0.0542	0.0142	0.0102	0.0124	0.0116
20	50	0.7	0.0684	0.0128	0.0104	0.0126	0.0120
20	50	0.8	0.1052	0.0130	0.0128	0.0154	0.0136
20	50	0.9	0.1880	0.0128	0.0250	0.0246	0.0252
20	100	0.5	0.0382	0.0210	0.0118	0.0164	0.0144
20	100	0.6	0.0516	0.0150	0.0108	0.0132	0.0110
20	100	0.7	0.0810	0.0158	0.0112	0.0152	0.0136
20	100	0.8	0.1258	0.0162	0.0142	0.0202	0.0178
20	100	0.9	0.2256	0.0160	0.0184	0.0282	0.0254
20	200	0.5	0.0288	0.0208	0.0178	0.0188	0.0186
20	200	0.6	0.0370	0.0216	0.0178	0.0196	0.0182
20	200	0.7	0.0570	0.0182	0.0168	0.0186	0.0174
20	200	0.8	0.0738	0.0170	0.0158	0.0196	0.0178
20	200	0.9	0.1378	0.0192	0.0214	0.0288	0.0250

Table 6: The results of the standard scenario ($N = 100$, $d = 5$, $ccr = 1:1$, $\rho = 0.9$) with varying AUC's and varying case-control ratios for the comparison of the different approaches (Fig. 5).

ccr	AUC	MCP	Logit	WB,Rade	WB,Normal	WB,Unif
1:1	0.5	0.0274	0.0180	0.0160	0.0154	0.0152
1:1	0.6	0.0450	0.0202	0.0174	0.0184	0.0182
1:1	0.7	0.0580	0.0204	0.0176	0.0200	0.0194
1:1	0.8	0.0774	0.0182	0.0170	0.0192	0.0192
1:1	0.9	0.1380	0.0212	0.0250	0.0290	0.0276
1:2	0.5	0.0312	0.0218	0.0164	0.0192	0.0180
1:2	0.6	0.0436	0.0176	0.0138	0.0164	0.0160
1:2	0.7	0.0492	0.0146	0.0122	0.0138	0.0134
1:2	0.8	0.0920	0.0178	0.0150	0.0194	0.0194
1:2	0.9	0.1520	0.0226	0.0262	0.0306	0.0302
1:4	0.5	0.0324	0.0220	0.0136	0.0178	0.0154
1:4	0.6	0.0386	0.0224	0.0188	0.0200	0.0202
1:4	0.7	0.0622	0.0262	0.0204	0.0236	0.0234
1:4	0.8	0.0806	0.0276	0.0240	0.0278	0.0266
1:4	0.9	0.1226	0.0276	0.0312	0.0350	0.0340
1:9	0.5	0.0470	0.0306	0.0176	0.0246	0.0214
1:9	0.6	0.0818	0.0390	0.0196	0.0310	0.0264
1:9	0.7	0.1382	0.0638	0.0370	0.0514	0.0456
1:9	0.8	0.1836	0.0840	0.0622	0.0722	0.0684
1:9	0.9	0.2050	0.0698	0.0688	0.0752	0.0704

Table 7: The results of the standard scenario ($N = 100$, $d = 5$, $ccr = 1:1$, $\rho = 0.9$) for ordinal data with varying AUC's for the comparison of the different approaches (Section 3.1).

AUC	MCP	Logit	WB,Rade	WB,Normal	WB,Unif
0.5	0.0294	0.0234	0.0196	0.0220	0.0212
0.6	0.0358	0.0218	0.0192	0.0216	0.0202
0.7	0.0446	0.0236	0.0202	0.0216	0.0206
0.8	0.0168	0.0044	0.0042	0.0044	0.0046
0.9	0.0810	0.0170	0.0142	0.0156	0.0152

Table 8: S8: The results of the power simulation for the comparison of the different approaches for the following scenario: $N = 200$, $d = 5$, $ccr = 1 : 1$, $\rho = 0.9$, $AUC = 0.7$ (Section 3.1).

ΔAUC	MCP	Logit	WB,Rade	WB,Normal	WB,Unif
0.00	0.0402	0.0222	0.0210	0.0204	0.0208
0.01	0.0648	0.0376	0.0352	0.0368	0.0362
0.02	0.1032	0.0660	0.0634	0.0652	0.0640
0.03	0.1672	0.1138	0.1100	0.1116	0.1126
0.04	0.2604	0.1900	0.1832	0.1872	0.1854
0.05	0.3590	0.2758	0.2704	0.2746	0.2700
0.06	0.4876	0.3888	0.3798	0.3814	0.3826
0.07	0.5994	0.5088	0.4972	0.5048	0.5012
0.08	0.7200	0.6284	0.6196	0.6244	0.6180
0.09	0.8080	0.7340	0.7282	0.7344	0.7304
0.10	0.8874	0.8362	0.8296	0.8330	0.8320
0.11	0.9504	0.9168	0.9124	0.9144	0.9132
0.12	0.9734	0.9518	0.9490	0.9500	0.9494
0.13	0.9886	0.9784	0.9762	0.9792	0.9776
0.14	0.9960	0.9904	0.9894	0.9898	0.9896
0.15	0.9988	0.9974	0.9972	0.9974	0.9974
