# Supplementary figures and images for: A Wild Bootstrap approach for the selection of biomarkers in early diagnostic trials
Source: BMC Med Res Methodol. 2015 May 1;15:43. doi: 10.1186/s12874-015-0025-y (PMC4426186; doi:10.1186/s12874-015-0025-y)

Empirical type-I error

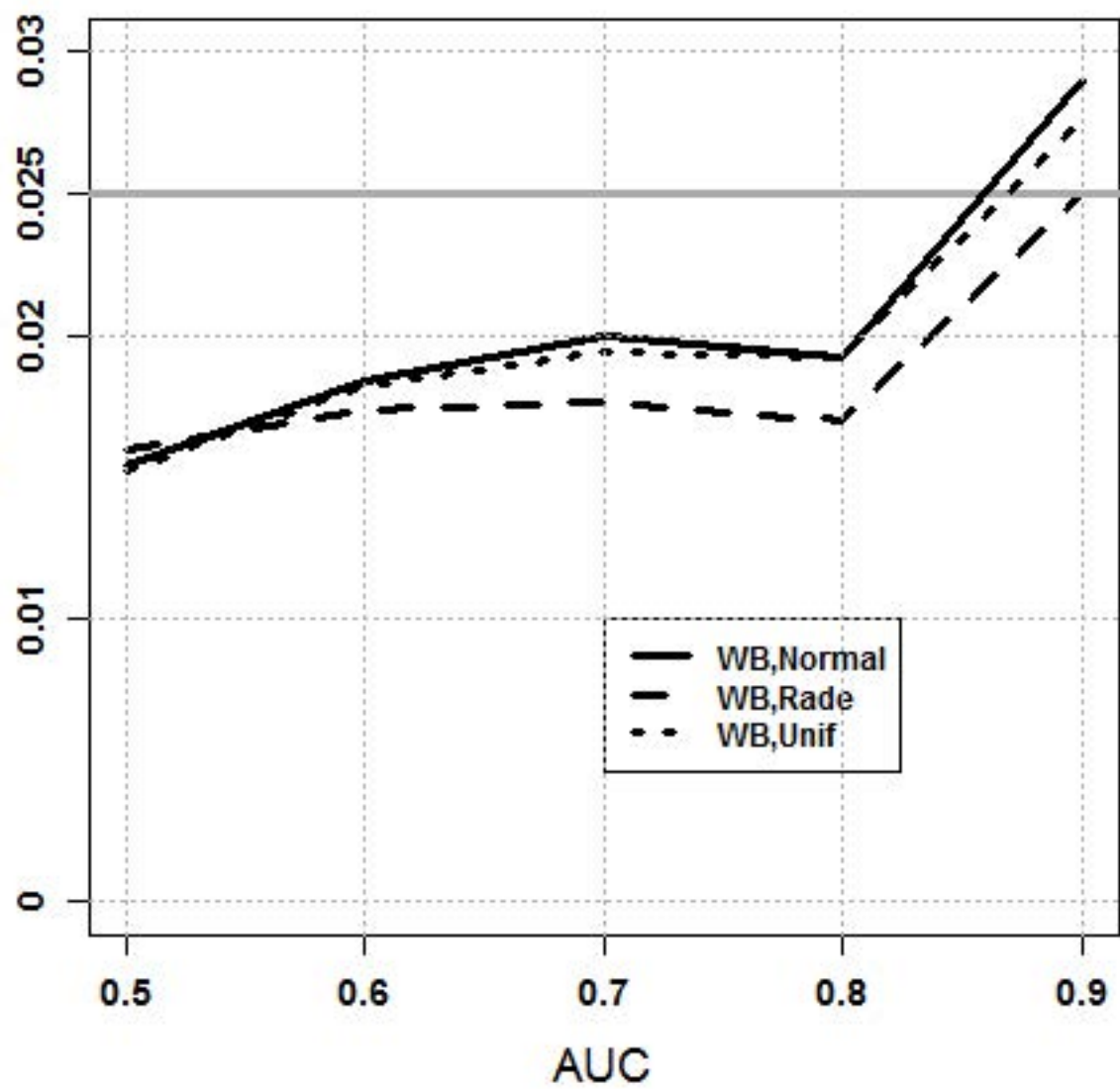

Supplement: Additional file 3 — Figure S1. Empirical type-I error of the Wild Bootstrap approach with the three different weights for the standard scenario (see article, Section “Simulation results”) with varying AUC’s. [file 12874_2015_25_MOESM3_ESM.pdf]

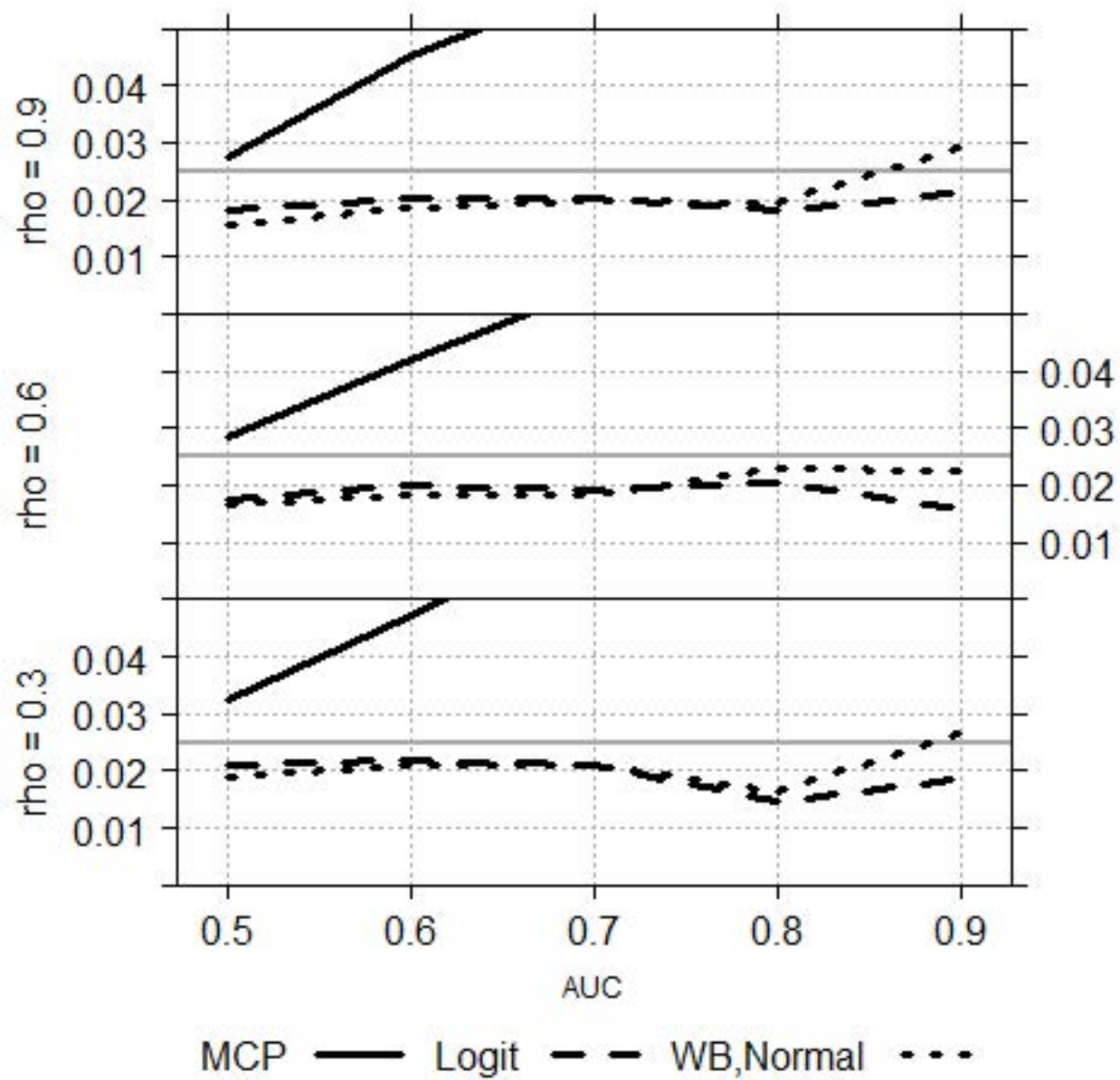

Supplement: Additional file 4 — Figure S2. Empirical type-I error of the MCP, the Logit and the WB-Normal approach for varying strength of correlation. [file 12874_2015_25_MOESM4_ESM.pdf]

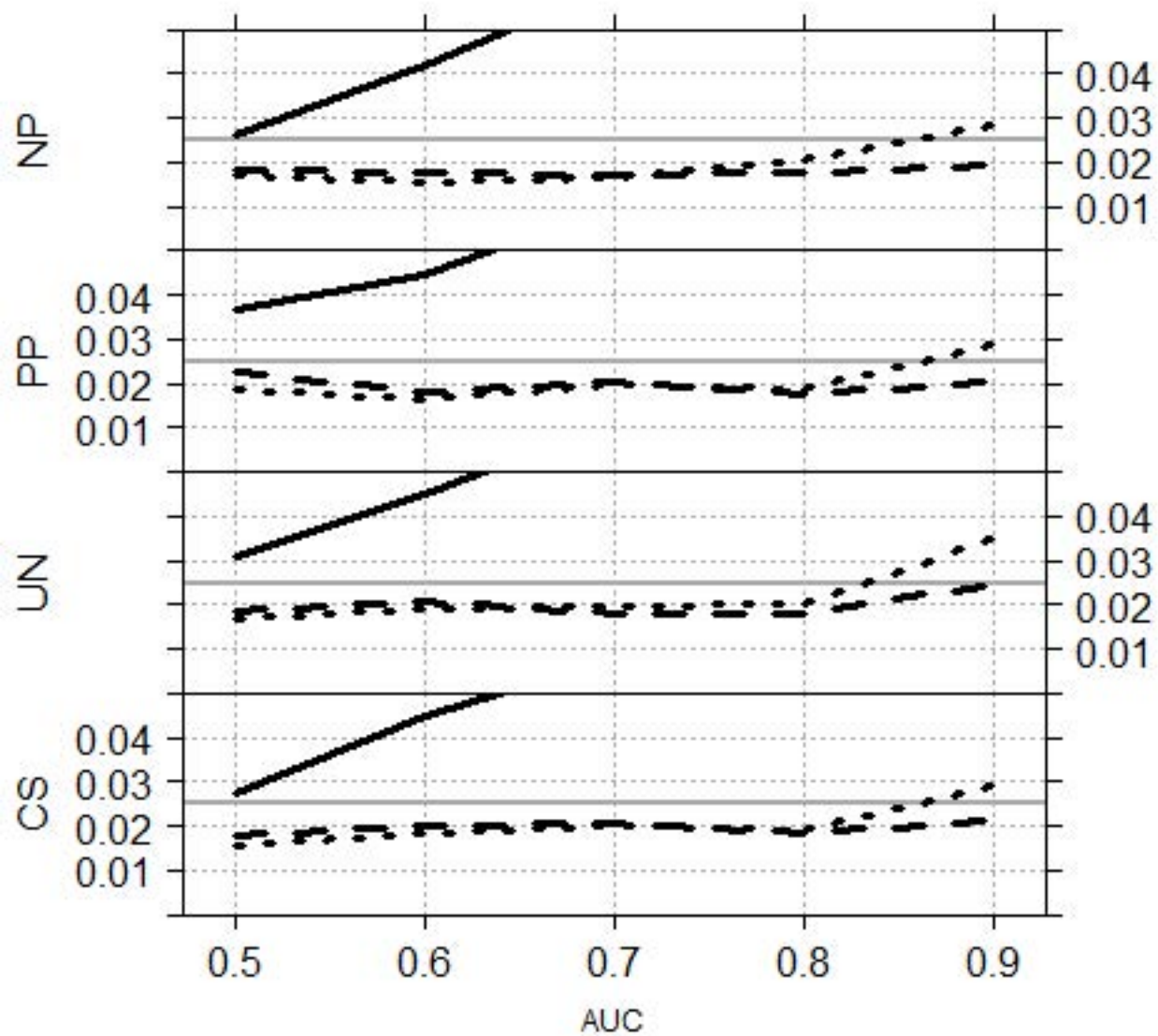

MCP ——— Logit - - - WB,Normal . . .

Supplement: Additional file 5 — Figure S3. Empirical type-I error of the MCP, the Logit and the WB-Normal approach for different covariance structures (CS: compound symmetry, UN: unstructured, PP/NP: diagonal matrix with heterogeneous variances and positive/negative pairing). [file 12874_2015_25_MOESM5_ESM.pdf]

Log Normal

Normal

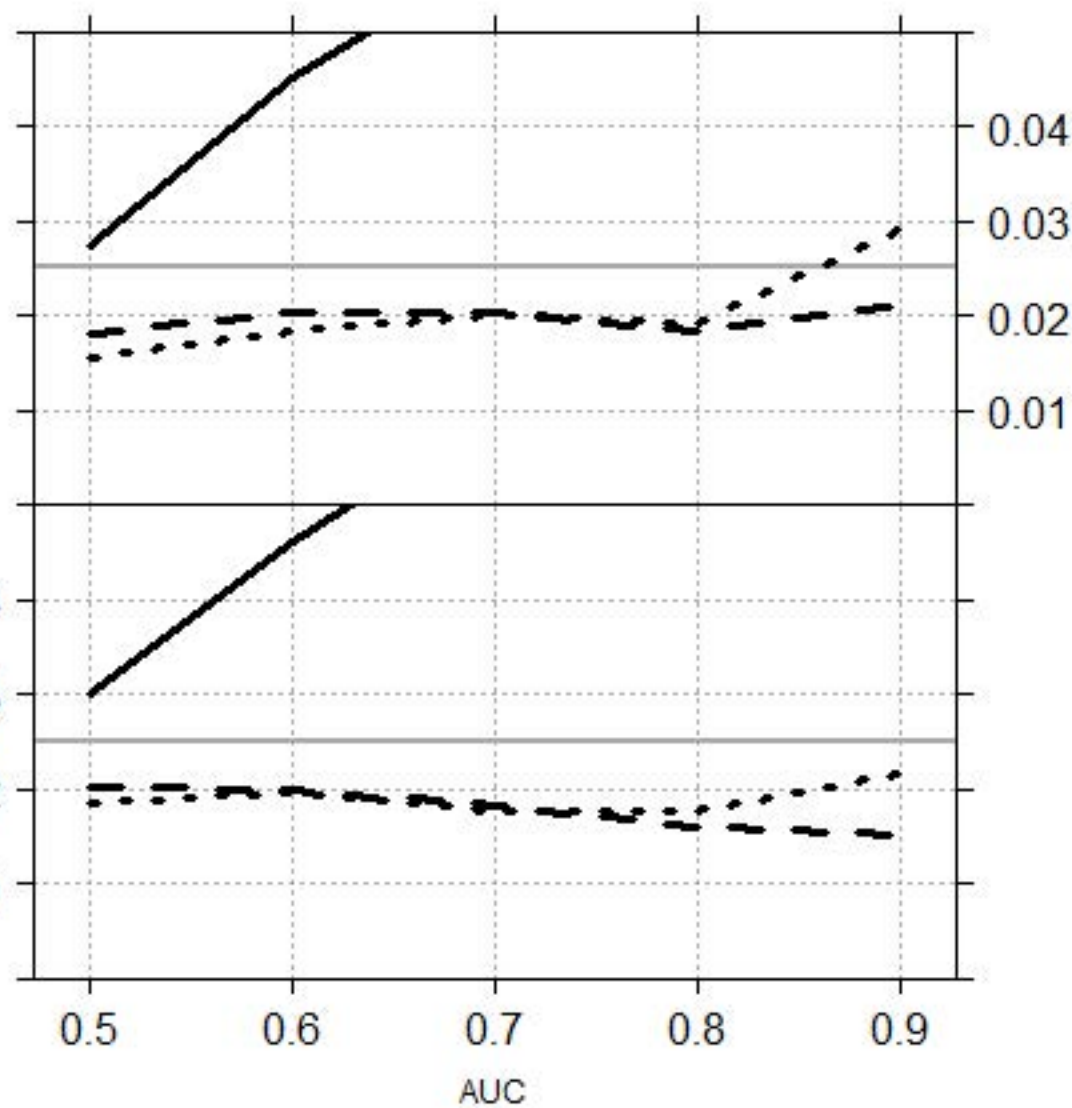

MCP — Logit - - - WB,Normal . . .

Supplement: Additional file 6 — Figure S4. Empirical type-I error of the MCP, the Logit and the WB-Normal approach for normal and log-normal distributed data. [file 12874_2015_25_MOESM6_ESM.pdf]
